# A Quality Improvement Approach to Sustainable Student-Led Leadership Development in Undergraduate Medical Education

**DOI:** 10.7759/cureus.102970

**Published:** 2026-02-04

**Authors:** Bryan J Fisher, J. Michael Overton

**Affiliations:** 1 Medicine, Florida State University College of Medicine, Tallahassee, USA

**Keywords:** emotional intelligence, leadership development, quality improvement, student-led programs, undergraduate medical education

## Abstract

Background

Leadership training is increasingly recognized as an important component of medical education, yet programs remain highly variable in delivery. The Leadership in Medicine (LIM) program at the Florida State University College of Medicine is a four-year, student-led leadership development program. As the only of its kind, LIM offers autonomy and hands-on training but faces challenges in consistency and sustainability.

Objective

The objectives of this study are to (1) evaluate LIM program weaknesses through an internal needs assessment and (2) apply plan-do-study-act (PDSA)-guided quality improvement (QI) interventions to address shortcomings. This study focuses on QI design and implementation; future evaluation through follow-up surveys and competency assessments aligned with the emotional and social competency inventory (ESCI) model will determine the effectiveness of these interventions.

Methods

A mixed-methods internal needs assessment (n = 21, 52.5% response rate) evaluated participant perceptions of key program elements. Quantitative data were analyzed using four-point Likert scales, while qualitative themes guided the QI intervention design.

Results

Participants noted deficiencies in program clarity (2.52/4), curriculum structure (2.55/4), and organizational consistency (2.57/4). Students expressed strong interest in poster presentation opportunities (85.7%) and leadership competency certificates (76.1%). Qualitative themes revealed limited curricular visibility, inconsistent transitions, and unclear expectations.

Intervention

Three QI domains were implemented: (1) structural enhancement through voting, a program roadmap, and meeting documentation; (2) sustainability through standardized transitions and annual budgeting; and (3) professional development through poster presentations, microlearning, and outside lectures. All interventions received unanimous approval from the managing cohort and executive board.

Conclusions

This intervention demonstrates how QI principles can be used to address shortcomings in the structure and autonomy of student-led leadership programs in undergraduate medical education. The implemented interventions are aimed at overcoming program areas for improvement and providing a framework for future evaluation. This reform proposes a feasible, adaptable model with the potential to enhance leadership development through evidence-based interventions.

## Introduction

Leadership is increasingly recognized as a fundamental competency for physicians, essential for making clinical decisions, navigating complex healthcare systems, collaborating within interprofessional teams, and driving system-level improvement. Despite this recent prioritization, leadership education during medical training remains inconsistent, with significant variability by program. Many medical schools lack leadership curricula, and those that do exist are often short-term or elective, offering limited longitudinal development [1,2].

At the Florida State University College of Medicine (FSU COM), a voluntary para-curricular leadership training program called “Leadership in Medicine” (LIM) was established to enhance leadership development for a select cohort of medical students in the FSU COM MD program. LIM is based on a four-year, student-led, and faculty-supported model with yearly select focuses to supplement the MD curriculum. LIM is unique among medical schools in that it is student-led. While 54% of medical schools offer a leadership development program, only one other school reports having a student-led leadership development program [3]. LIM’s leadership development curriculum is based on the emotional and social competency inventory (ESCI) model, developed by Richard Boyatzis and Daniel Goleman [4]. By structuring LIM around this model, the program provides students with a language and framework for self-assessment and longitudinal leadership growth throughout the full course of their undergraduate medical training.

While the ESCI framework provides a strong theoretical foundation, LIM’s implementation has revealed opportunities for improvement in maintaining longitudinal consistency and structural continuity. Due to the student-led nature of the program, variability in leadership transitions, inconsistent documentation, and unclear curricular expectations potentially jeopardize program continuity across cohorts and limit the educational value and professional development outcomes for participating students. Addressing these structural shortcomings through competency-based frameworks and evidence-informed quality improvement (QI) is essential for enhancing LIM’s impact.

Problem statement

LIM’s unique four-year structure presents an opportunity to serve as a national model for integrating leadership training into undergraduate medical education (UME) without requiring a formal dual degree. By enhancing LIM’s structure, visibility, and sustainability through evidence-based QI measures, this project contributes to the field by demonstrating how student-led and para-curricular leadership programs can be strengthened using internal needs assessments and competency-based frameworks. This QI design and implementation study aims to address program weaknesses through targeted interventions, in which future evaluation through surveys and ESCI competency assessments will assess the effectiveness of these interventions.

## Materials and methods

Study design

This project is a initiative designed to enhance the structure, sustainability, and professional development value of the LIM program at FSU COM. Guided by the ESCI framework as well as principles of continuous improvement, this project aims to (1) strengthen LIM’s organizational infrastructure, (2) align program offerings with student needs and national competency standards, and (3) increase the tangible professional benefits of student participation. The data collection and intervention design phase took place between May and September 2025, with full implementation of program reforms planned for the 2025-2026 academic year.

This project was developed utilizing the plan-do-study-act (PDSA) model of QI. In the “plan” phase, program needs were identified utilizing a needs assessment. The “do” phase consisted of implementing a set of interventions targeting identified shortcomings. Implementation voting and initial feedback represent the “study” phase, and the “act” phase involves ongoing monitoring through annual all-cohort surveys and iterative policy adjustments informed by those data. Future PDSA cycles will occur with post-implementation LIM all-cohort surveys, the first of which will be conducted in May 2026. The 2026 LIM all-cohort survey will serve as the primary endpoint for the first PDSA cycle. This framework ensures continuous refinement of the LIM program in alignment with student feedback and institutional goals.

Needs assessment

To identify strengths and areas for improvement, a comprehensive needs assessment was conducted via the 2025 LIM all-cohort survey. This internal evaluation was distributed to all active LIM participants (n = 40) across the four program cohorts via a voluntary, anonymous electronic form. A total of 21 LIM participants completed the survey (52.5% response rate). Inclusion criteria consisted of current enrollment in the LIM program during the 2025 academic year; students not participating in LIM or those who did not respond to the survey were excluded. The only demographic variable collected was cohort year. The survey was administered in May 2025, and participants were informed that responses were voluntary and de-identified. The questionnaire included both quantitative Likert-scale items and open-ended qualitative responses, enabling mixed-methods evaluation of student experience. Emergent themes informed the design of targeted QI interventions.

All questions from the 2025 LIM all-cohort survey can be found in Appendix A. Questions were a mix of items adapted from previous LIM surveys and newly developed items aimed at reflecting perceptions of program domains identified in prior leadership education literature. New items were reviewed by prior LIM managing cohort members to ensure relevance. Formal psychometric validation was not performed as the survey was designed for internal QI purposes rather than hypothesis testing.

Survey items were grouped into descriptive domains reflecting common areas of concern, including program clarity (as it relates to expectations, roles, timelines, and communication) and curriculum structure (as it relates to the organization and integration of LIM activities, as well as the curriculum itself). These domains were used to facilitate interpretation rather than to represent formally validated constructs. While these domains may overlap, they capture distinct aspects of participants' experience with the LIM program.

Quantitative and qualitative analysis

Quantitative survey data were summarized using means and standard deviations for each Likert-style item (1 = poor, 4 = excellent). To evaluate whether overall satisfaction with LIM differed across the four student cohorts, a one-way ANOVA was performed. In addition, paired *t*-tests were conducted to compare overall satisfaction with other program domains (clarity of expectations, curriculum structure, and organization of LIM). Paired analyses included only respondents who provided complete data for both items being compared. Statistical significance was defined as *p* < 0.05. All analyses were conducted using Microsoft Excel's Data Analysis ToolPak (Microsoft Corp., Redmond, WA).

Likert-style items are ordinal by design but treated as approximately interval and summarized using means and standard deviations, which is consistent with common practice in medical education QI studies. Statistical comparisons were intended to explore patterns and relative differences within the sample rather than to support causal or broadly generalizable claims.

Open-ended survey responses were analyzed using a descriptive thematic approach. Responses were reviewed by the study team to identify recurrent themes. Formal qualitative coding frameworks or software-assisted analysis were not employed, given the small-scale QI focus of the study.

Intervention design

Based on survey findings, the QI initiative was structured into three broad areas of improvement (domains): structural enhancement, long-term program sustainability, and professional development visibility.

Domain one: structural enhancement

**Figure 1:**
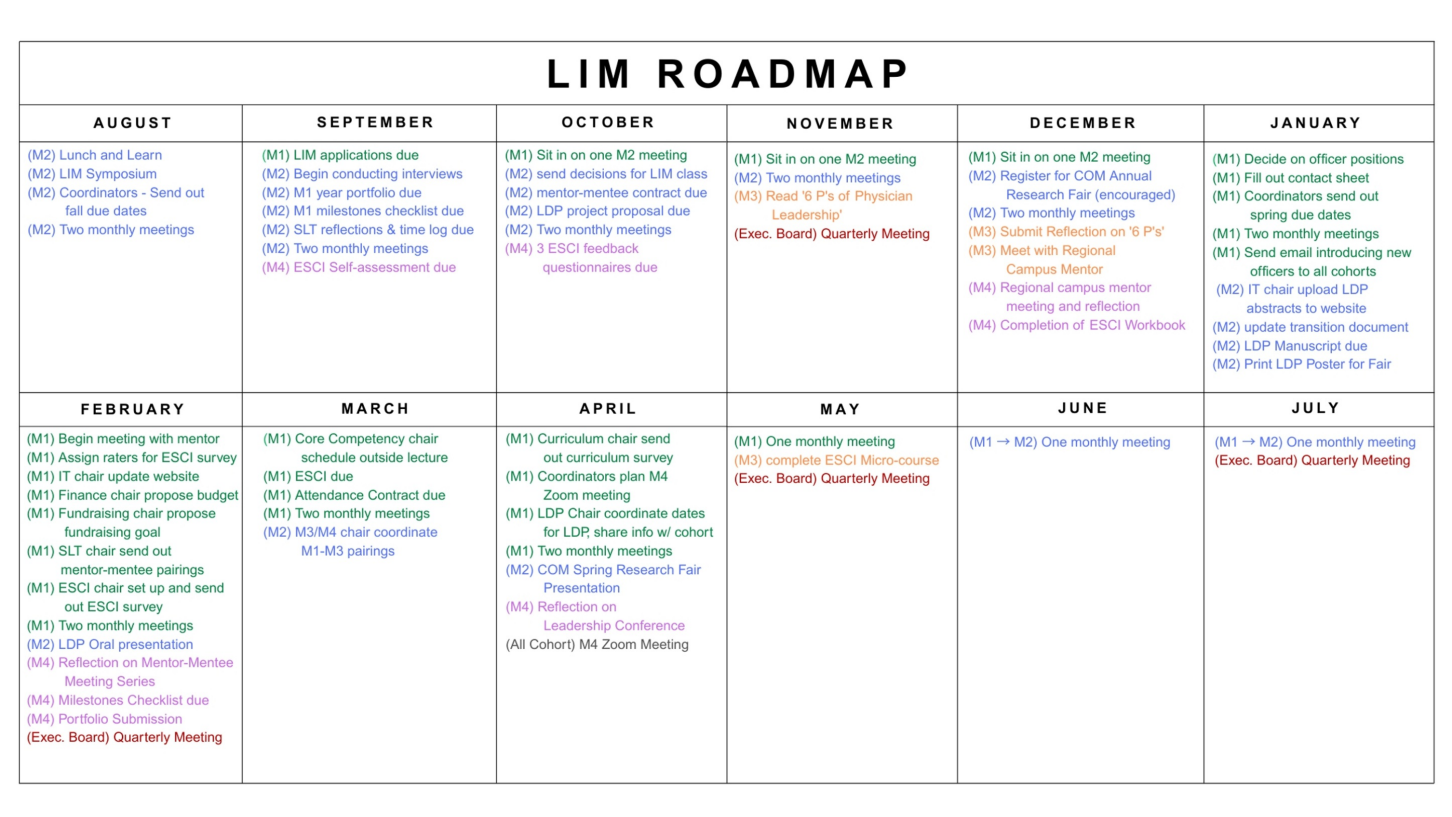
Month-by-month roadmap for the Leadership in Medicine program This roadmap outlines important timelines for roles, responsibilities, due dates, and expectations. Roadmap was placed into the LIM policies and procedures handbook available for all members, and the exact timeline is to be adapted by each year’s managing cohort. SLT: Specialized leadership training; LDP: Leadership development project; M1: First-year medical student; M2: Second-year medical student; M3: Third-year medical student; M4: Fourth-year medical student.

Designed to improve and implement structure into the program, these measures intend to enhance transparency and student engagement through a series of structured changes in the program’s policies and procedures handbook. Key elements of domain one include a formalized voting procedure, a month-by-month program “roadmap” (Figure 1), and a policy requiring meeting minutes to be taken at all LIM meetings.

Domain two: long-term program sustainability

These measures ensure that improvements to LIM persist beyond the current academic year by addressing leadership continuity and financial oversight. Measures to increase program continuity include developing standardized role transition documents to outline responsibilities, timelines, and legacy notes for each role. Financial continuity will be promoted through annual budget reporting and fundraising goals proposed by their respective chairs.

Domain three: professional development and visibility

These initiatives are designed to enhance the visibility and real-world value of LIM participation. To do this, the program will coordinate LIM Leadership Development Project participation in FSU College of Medicine’s Annual Research Fair in the form of a poster presentation. Additionally, each LIM participant will complete a low-cost online microcourse mapped to their lowest ESCI domain during their third year of medical school (M3). To further improve professional development, at least one annual lecture will be required to be delivered by a physician or other community member addressing an ESCI competency identified as an area of need.

Implementation of interventions

Proposed interventions were drafted by the study lead and reviewed by the LIM managing cohort, executive board, and the program's faculty advisor. An anonymous, simple majority approval from all parties was required for the implementation of the proposed interventions. Policies were formally incorporated into the LIM Student Handbook and enacted beginning in January 2026.

Evaluation plan

The impact of the QI interventions will be evaluated through follow-up surveys administered post-implementation (to be conducted in the 2026 all-cohort survey and beyond, modeled after the 2025 all-cohort survey). This will include qualitative analysis of free-response feedback as well as quantitative comparisons of pre- and post-implementation average Likert ratings on items related to structure, expectations, satisfaction, and leadership growth.

Institutional Review Board (IRB) and ethics

This study received a determination of "Not Human Subjects Research" from the Florida State University IRB, as it involved secondary analysis of de-identified QI data (FSU IRB#STUDY00006436).

## Results

Results of 2025 LIM all-cohort survey

A total of 21 LIM members (52.5% of program participants) completed the survey, including representation from all four cohorts. Respondent demographics, limited to cohort year, are provided in Table 1.

**Table 1:** Demographic characteristics of survey respondents The cohort year distribution of survey respondents is shown as both absolute counts and percentages of the total sample. M1: First-year medical student; M2: Second-year medical student; M3: Third-year medical student; M4: Fourth-year medical student.

Cohort Year	Number of Respondents (n)	% of Respondents
2028 (M1)	9	42.90%
2027 (M2)	7	33.30%
2026 (M3)	2	9.50%
2025 (M4)	3	14.30%
Total	21	100%

Quantitative results utilizing the four-point Likert scale [5] (1 = poor, 4 = excellent) revealed that overall satisfaction with LIM was adequate (M = 3.10, SD = 0.70). However, several program areas scored lower, including “clarity of program expectations” (M = 2.52, SD = 1.07), which was significantly lower than overall satisfaction (*t*(20) = -3.01, *p* = 0.007). “Curriculum structure” (M = 2.55, SD = 0.75) was also rated lower than overall satisfaction, *t*(19) = -2.98, *p* = 0.007. Similarly, “organization of LIM” (M = 2.57, SD = 1.02) was rated lower than overall satisfaction, *t*(20) = -2.75, *p* = 0.012. When stratifying results across cohorts, overall satisfaction with LIM was highest among second-year students (M = 3.28) and lowest among fourth-year students (M = 2.67), suggesting a perceived decline in structure and communication over time; however, these differences were not statistically significant, *F*(3,17) = 0.52, *p* = 0.674. These cohort-level comparisons, along with paired analyses of key program domains, are summarized in Table 2.

**Table 2:** Statistical comparison of key program ratings Mean Likert scores range from 1 (poor) to 4 (excellent). One-way ANOVA was used to compare overall satisfaction across cohorts (M1-M4). Paired *t*-tests were conducted to compare overall satisfaction with other program domains. Only respondents with complete data were included in each paired test. Asterisks indicate statistically significant differences. M1: First-year medical student; M4: Fourth-year medical student.

Survey Item/Comparison	Mean (SD)	Statistical Test Type	Test Statistic	*p*-value
Overall satisfaction (M1–M4)	3.10 (0.70)	One-way ANOVA	*F*(3,17) = 0.52	0.674
Clarity of expectations vs. overall satisfaction	2.52 (1.07) vs. 3.10 (0.70)	Paired *t*-test	*t*(20) = -3.01	0.007*
Curriculum structure vs. overall satisfaction	2.55 (0.75) vs. 3.10 (0.70)	Paired *t*-test	*t*(19) = -2.98	0.007*
Organization of LIM vs. overall satisfaction	2.57 (1.02) vs. 3.10 (0.70)	Paired *t*-test	*t*(20) = -2.75	0.012*

In addition to quantitative items, open-ended responses offered further insights into LIM members’ experiences and areas for improvement. Thematic analysis identified three recurring issues: insufficient program clarity, inconsistent continuity across cohorts, and limited curricular organization and visibility. Themes were found to be similar across cohorts, though certain emphases regarding a lack of program expectations were more frequently articulated by first-year students. This pattern supported the development of program-wide interventions rather than ones focused on supporting an individual cohort.

Representative comments from survey participants are given in Table 3.

**Table 3:** Themes identified from qualitative survey responses The representative quotes were extracted from open-ended survey responses and grouped into thematic categories following an inductive qualitative analysis approach. M1: First-year medical student.

Theme	Representative Quote
Lack of program structure	“LIM just needs more structure, especially in the first year. The M1 cohort is unsure of what they are supposed to do during their meetings.… I think having a detailed calendar/syllabus of events for the M1 year would help the new leading cohort figure out how to manage their time and will help them understand the tasks that need to be accomplished.”
Unclear expectations and curriculum visibility	“I think that I'm learning more about the curriculum as we go along instead of having a broad idea from day 1.”
Need for clearer timelines and organization	“It would be helpful to have a clear timeline of assignments, especially explaining the assignments across all 4 years.”
Desire for improved meeting organization	“Possible meeting agendas and meeting minutes being recorded?”
Limited external guidance/Desire for mentorship	“Outside help is super necessary—blind leading the blind model is great for learning quickly, but it would be nice to learn from people who have led in their communities their whole lives.”
Curricular improvement and assignment variety	“More variety in assignments, there are just a lot of reflections.”

These results directly informed the design of interventions within the structural enhancement, program sustainability, and professional development domains.

**Figure 2:**
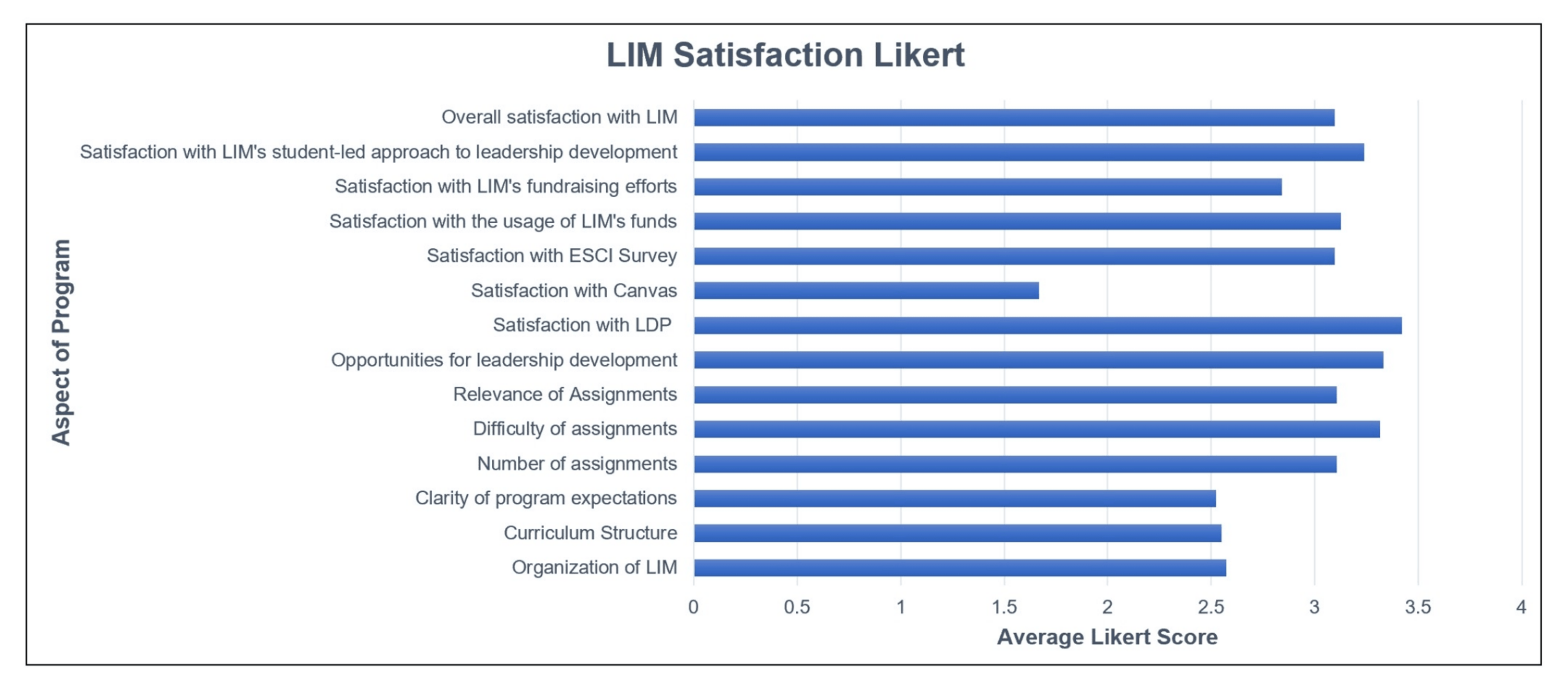
Likert-style question results The participants were asked to rate different aspects of the LIM program on a scale of poor (1), fair (2), good (3), excellent (4), or not applicable. LIM: Leadership in Medicine.

Interest in new initiatives was also gauged using the survey. A list of nine potential initiatives was listed, as well as an “other - fill in” response. These results are shown in Figure 2. Of the listed options, the five highest rated were “opportunity for poster presentation of LDP at COM or local conference” (85.7% in favor), “networking events with physician leaders” (85.7%), “outside lectures from physician and community leaders” (76.1%), “certificates of completion for leadership competency training” (76.1%), and “CV/LinkedIn/resume building workshop” (76.1%).

**Figure 3:**
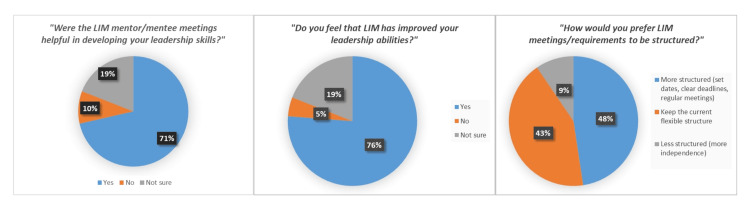
Multiple-choice question results Results of the three multiple-choice questions in the 2025 LIM all-cohort survey, illustrating student preferences for new program initiatives.

Three more questions, gauging perception of mentorship, leadership development, and program structure, were also included in the survey. Perception of mentorship and leadership development from LIM was generally high, but structure was lacking, with most participants saying they would either want to keep or increase the amount of structure in the program. Only 9.5% of participants said they would prefer less structure in LIM. The results of these questions are shown in Figure 3.

Following the development of interventions, the management of the cohort and executive board votes were held to approve the adoption of program changes. All proposals received unanimous support from participating members, which suggests strong engagement and perceived value of the planned improvements.

## Discussion

Interpretation of key findings

The findings of this QI initiative highlight both the strengths and areas for improvement within the LIM program. Students reported high satisfaction with leadership opportunities, mentoring, and project-based learning but identified persistent issues with program clarity, continuity, and curricular organization. These themes directly informed the design of the QI initiatives. Early engagement (evident by the unanimous cohort support for implementation) suggests there is a strong perceived value of these interventions. The goal of these interventions is to address program shortcomings identified in the survey; however, program perception post-intervention is yet to be assessed.

Initiatives for structural enhancement

Based on the low-scoring domains in the 2025 all-cohort survey of curriculum structure and clarity of program expectations, three policies were developed and voted into the LIM Student Handbook: (1) a formal voting policy to ensure transparent decision-making and cohort-wide participation in all program and curricular changes, (2) a month-by-month LIM roadmap integrated into the handbook to outline key meetings and milestones, and (3) a note-taking and documentation policy to preserve institutional knowledge across leadership transitions.

Structured governance frameworks in higher education have been proven to lead to better-quality decisions and sustained adoption of curricular change [6]. Recent syntheses in higher education show that shared governance works when rules for participation and voting are explicit and transparent; unclear processes erode trust and stall implementation [7]. Curriculum mapping is a foundational med-ed strategy to improve program efficiency and clarity. AMEE (Association for Medical Education in Europe) Guide No. 21 [8] emphasizes that mapping helps improve transparency and reduce ambiguity. Integrating a month-by-month roadmap is a direct application of this guidance. Knowledge management (KM) literature in higher education identifies institutional memory as critical to continuity. KM frameworks specifically recommend routine capture of meeting notes, decisions, and “how-to” procedures to maintain quality and organizational learning over time [9].

Initiatives for program sustainability

The second domain of improvement focused on the long-term sustainability of the LIM program through standardized leadership transitions and financial accountability. Though LIM’s student-led model promotes autonomy, it also introduces inherent instability due to annual leadership turnover and the absence of formalized continuity systems. Qualitative responses from the 2025 all-cohort survey identified inconsistent transitions and a lack of clear roles and responsibilities as common themes. Two initiatives were developed to address these challenges: (1) a transition policy requiring each outgoing chair to update standardized transition documents, including descriptions of responsibilities, deadlines, and “legacy notes” and (2) a budget and spending policy establishing an annual spending plan, fundraising goals, and expenditure tracking mechanisms.

Turnover is a known instability point in student-run programs. Reviews of student-run free clinics (the closest analog to student-governed programs in UME) repeatedly flag sustainability risks from annual leadership changes, recommending formal structures and documentation to maintain continuity [10].

An internal financial analysis conducted in August 2025 revealed that LIM’s expenditures are outpacing fundraising. Discussion among LIM members showed that LIM’s financial outlook was poorly understood and of a lower priority. Integrating a budget and spending policy directly addresses these findings, allowing future cohorts to be more mindful of spending and fundraising efforts. An annual budget with predefined categories as well as fundraising targets aligns with evidence that financial transparency mechanisms improve stewardship and stakeholder engagement [11] as well as match prevailing university standards [12].

Initiatives for professional development and visibility

Open-ended survey questions identified the disproportionate number of reflection-style assignments as an area for program improvement. Additionally, 85.7% of participants were interested in an opportunity to present the Leadership Development Project (LDP) in poster form, and 76.1% of participants were interested in seeing certificates of completion for leadership competency training. Thus, three policies were made to improve professional development and visibility: (1) poster presentation policy, recommending that all LIM LDPs be presented at the FSU College of Medicine Annual Research Fair; (2) a curriculum revision introducing microlearning modules, wherein each student completes a short online leadership course mapped to their lowest ESCI domain; and (3) an outside lecture policy, requiring at least one lecture a year be delivered by a physician or community leader addressing an ESCI competency identified as an area of need.

Scholarly dissemination via posters has been shown to improve students’ science identity and science communication self-efficacy [13]. An increased number of research publications (in which poster presentations are counted) is also proven to correlate with better residency match outcomes [14-16]. Microlearning interventions are increasingly utilized in healthcare continuing education [17] and have demonstrated efficacy in improving engagement and skill acquisition [18]. Invited physician-lecturers bring external legitimacy and authenticity to leadership content, bridging the theory-to-practice divide and reinforcing the relevance of ESCI competencies.

Relationship to previous research and literature

The findings from this project reinforce previously reported gaps in UME leadership curricula. Prior literature [1,2] has identified variability in program structure and lack of standardized frameworks as areas for improvement. The LIM baseline (2025 all-cohort) survey results mirrored these deficiencies at the program level, revealing inconsistent expectations, role ambiguity, and limited curricular visibility as opportunities for growth. Rather than simply confirming these trends, this project aimed to implement evidence-based interventions to address the low-scoring domains of the baseline survey.

Existing UME leadership programs described in the literature are primarily faculty-driven, short-term, and resource-intensive, if not a dual-degree MBA program offered in concurrence with the MD or DO degree [19,20]. These existing UME leadership programs often fail to achieve sustainability and effectiveness due to limited student engagement and inadequate institutional bandwidth.

The LIM program at FSU College of Medicine operationalizes leadership training into a student-governed, competency-based, longitudinal program supported by minimal faculty oversight. By leveraging the ESCI model and continuous program evaluation, LIM demonstrates that longitudinal, student-led leadership development can be both scalable and self-sustaining within the existing UME structure. This resulting model directly addresses calls in recent literature for reproducible, longitudinal UME leadership programs that can self-sustain with student ownership [1,2].

In addition, the incorporation of QI methodology into UME leadership training is a novel contribution to the literature. Few published models employ systematic feedback loops to guide curricular reform over time. Studies applying QI-based frameworks in other areas of medical education have demonstrated the effectiveness of needs-assessed, continuously refined curricula [21].

Implications for LIM program redesign

The results of this project extend prior findings by demonstrating how student-led programs can operationalize best practices from recent leadership education reviews through a continuous QI framework. Prior literature [1,2] has called for sustainable, competency-based leadership models that persist over time, and the findings from this study support those conclusions. By implementing targeted, evidence-informed interventions, this project demonstrates how common student-led program limitations (clarity, continuity, and visibility) can be effectively addressed within existing institutional and resource constraints.

While many published leadership programs remain faculty-led and resource-intensive, the approach described here underlines the feasibility of student-led program improvement by internal needs assessment. Rather than introducing a new methodology, this project applies well-established QI principles to bridge the gap between leadership theory and its practical implementation in UME.

These findings extend existing literature by offering a pragmatic example of how medical schools can implement (and continuously seek improvement of) para-curricular leadership education without requiring new degree programs or significant funding. In doing so, this project contributes an adaptable framework that other UME leadership initiatives can use to enhance structure, sustainability, and professional visibility within their own programs.

Some of the findings within this study are broadly transferable, likely applicable to programs operating in different contexts than LIM. In student-led programs, challenges related to program clarity and organizational continuity are commonly reported, especially in programs without substantial institutional resources. Likewise, the observed value of transparent governance mechanisms is broadly applicable to most organizational structures. Measures such as voting, note-keeping, standardized transitions, and microlearning have been well-described within the literature to have improved decision-making, program continuity, and educational value [6,9,18]. Additionally, applying a PDSA approach with an internal needs assessment followed by targeted interventions represents a transferable process that can be applied in different contexts.

These interventions were planned for usage in the context of a student-led, faculty-supported leadership development program; however, policies can be adapted to a faculty-led leadership development program, as core principles of program continuity, shared governance, professional development, and needs-informed QI design are broadly transferable. More nuanced and structured implementation may be required in these settings, especially if there is already a strong organizational framework in place.

In contrast, some of the findings are context-dependent, including many of the more specific survey questions and their answers, as well as the implementation of certain initiatives that are influenced by local factors (e.g., poster presentation integration and external lectures). Additionally, financial pressures are context-dependent, and solutions depend on organizational regulations and available funding mechanisms. While establishing a budget is generally a prerequisite measure, other interventions are dependent on specific institutional conditions.

Limitations and future directions

This study was conducted within a single medical school, with a cohort of 40 participants, in which 52.5% (n = 21) of participants took part in the needs assessment. Given the small sample size, the study may not be powered to support broad statistical inference. As such, the findings may reflect the unique institutional culture and leadership emphasis at FSU COM rather than representing all UME environments. Furthermore, the data were self-reported and cross-sectional, capturing perceptions at a single time point before the implementation of reforms. As participation in the survey was voluntary, the potential for selection bias exists, where students who were more engaged or more dissatisfied with the program may have been more likely to respond to the survey. Thus, it is important to note that the results of the survey may not fully represent the perspective of all LIM participants.

Methodological limitations should also be acknowledged. The LIM all-cohort survey was internally developed for program evaluation purposes and not formally validated, which may affect measurement reliability and transferability. The rigor of the qualitative analysis was restricted by the use of a thematic approach instead of formal coding. Additionally, quantitative analyses utilized parametric statistical tests on Likert-scale data, an approach frequently employed in medical education research but associated with known methodological limitations.

The student-led nature of the LIM program introduces inherent variability in program delivery due to the constant turnover of managing cohorts. Though the QI initiatives in this project hope to improve continuity and structure, their effectiveness is dependent on future LIM cohorts to adhere to newly established policies and the maintenance of documentation. Continued faculty oversight and yearly review via the curriculum chair will be essential to ensure that the implemented reforms maintain their alignment with the program’s long-term needs and objectives.

These limitations are consistent with early-stage, single-institution QI studies. Future PDSA cycles will incorporate post-implementation surveys, process measures (such as participation and documentation completion metrics), and longitudinal assessments of ESCI competencies to evaluate the effectiveness of the program redesign across cohorts.

## Conclusions

This project describes how QI principles can be applied to student-led leadership programs in UME to address gaps in program structure, sustainability, and professional development value. With guidance from programmatic feedback and alignment with the ESCI framework, the implemented initiatives are designed to address key program shortcomings (structure, sustainability, and visibility) while maintaining autonomy, a vital component of the LIM program at FSU COM.

This reform proposes a practical framework that may be adaptable to other institutions seeking to promote student ownership in medical leadership education. Although the long-term outcomes of these interventions are yet to be assessed, early interest and unanimous program approval suggest there is strong stakeholder engagement and feasibility for continued program success. Future evaluation through longitudinal internal program surveys and competency assessments will be helpful to determine whether these reforms lead to measurable gains in program satisfaction and leadership growth. The potential to use continuous QI principles to create a sustainable model for student-led para-curricular leadership growth in UME programs is highlighted in this work.

## Appendices

Appendix A: 2025 LIM all-cohort survey questionnaire

**Table 4:** 2025 LIM all-cohort survey questions This table lists all the questions used for the 2025 LIM all-cohort survey, a voluntary and anonymous needs assessment sent via email to all participants within the LIM program. The questionnaire included multiple-choice, Likert-style, multiple-answer, and free-response questions. LIM: Leadership in Medicine; LDP: Leadership Development Project; ESCI: Emotional and Social Competency Inventory; COM: College of Medicine. Source: Bryan J. Fisher and J. Michael Overton. This questionnaire is the original creation of the authors.

Query	Question Type	Answer Choices
"Which cohort are you part of?"	Multiple choice	2025; 2026; 2027; 2028
"Organization of LIM"	Likert, "How would you rate the following aspects of the LIM curriculum?"	Poor; Fair; Good; Excellent; Not Applicable
"Curriculum structure"	Likert, "How would you rate the following aspects of the LIM curriculum?"	Poor; Fair; Good; Excellent; Not Applicable
"Clarity of program expectations"	Likert, "How would you rate the following aspects of the LIM curriculum?"	Poor; Fair; Good; Excellent; Not Applicable
"Number of assignments"	Likert, "How would you rate the following aspects of the LIM curriculum?"	Poor; Fair; Good; Excellent; Not Applicable
"Difficulty of assignments"	Likert, "How would you rate the following aspects of the LIM curriculum?"	Poor; Fair; Good; Excellent; Not Applicable
"Relevance of assignments"	Likert, "How would you rate the following aspects of the LIM curriculum?"	Poor; Fair; Good; Excellent; Not Applicable
"Opportunities for leadership development"	Likert, "How would you rate the following aspects of the LIM curriculum?"	Poor; Fair; Good; Excellent; Not Applicable
"Satisfaction with Canvas"	Likert, "How would you rate the following aspects of the LIM curriculum?"	Poor; Fair; Good; Excellent; Not Applicable
"Satisfaction with ESCI survey"	Likert, "How would you rate the following aspects of the LIM curriculum?"	Poor; Fair; Good; Excellent; Not Applicable
"Satisfaction with the usage of LIM's funds"	Likert, "How would you rate the following aspects of the LIM curriculum?"	Poor; Fair; Good; Excellent; Not Applicable
"Satisfaction with LIM's fundraising efforts"	Likert, "How would you rate the following aspects of the LIM curriculum?"	Poor; Fair; Good; Excellent; Not Applicable
"Satisfaction with LIM's student-led approach to leadership development"	Likert, "How would you rate the following aspects of the LIM curriculum?"	Poor; Fair; Good; Excellent; Not Applicable
"Overall satisfaction with LIM"	Likert, "How would you rate the following aspects of the LIM curriculum?"	Poor; Fair; Good; Excellent; Not Applicable
"Were the LIM mentor/mentee meetings helpful in developing your leadership skills?"	Multiple choice	Yes; No; Not Sure
"Do you feel that LIM has improved your leadership abilities?"	Multiple choice	Yes; No; Not Sure
"Room for explanation of answer choices, if necessary."	Free response	N/A
"What would you change about the current LIM curriculum?"	Free response	N/A
"What would make the LIM assignments more applicable to your current year of training?"	Free response	N/A
"How would you prefer LIM meetings/requirements to be structured?"	Multiple choice	More structured (set dates, clear deadlines, and regular meetings); less structured (more independence); keep the current flexible structure
"What is the one thing you would add or remove from the curriculum?"	Free response	N/A
"Which initiatives would you be interested in seeing implemented in LIM? Please select all that apply."	Multiple answer	Opportunity for poster presentation of LDP at COM or local conference; switching from Canvas to Teams for assignments and calendar; outside lectures from physician and community leaders; certificates of completion for leadership competency training; shadowing opportunities with community/physician leaders; create your own LIM digital portfolio (showcasing your project, proficiencies, etc.); collaboration with College of Business' Healthcare MBA program; networking events with physician leaders; CV/LinkedIn/resume-building workshop
"Would you like to see formal training in any of these focus areas added to LIM's curriculum?"	Multiple answer	Public speaking; project management; networking and relationship building; advocacy and policy; time management
"Please share any additional feedback or suggestions to improve the LIM program."	Free response	N/A
